# Minimal Invasive Surgical Management of Familial Arteriovenous Malformation

**DOI:** 10.1155/2021/5564470

**Published:** 2021-06-09

**Authors:** Mokhtar Mamdouh Abdel-Latif, Shankargouda Patil

**Affiliations:** ^1^Maxillofacial Surgery and Diagnostic Sciences, Division of Oral Surgery, College of Dentistry, Jazan University, Jazan, Saudi Arabia; ^2^Maxillofacial Surgery and Diagnostic Sciences, Division of Oral Pathology, College of Dentistry, Jazan University, Jazan, Saudi Arabia

## Abstract

**Introduction:**

Familial arteriovenous malformations are exceedingly rare. They are often noted at birth. They can also present during childhood or adolescence. Sclerotherapy has proven to have a favorable outcome. *Case Presentation*. The present case report describes the treatment of arteriovenous malformations on the tongue, labial mucosa, and vermilion border in siblings treated with boiling saline injections.

**Conclusion:**

Sclerotherapy using boiling saline had shown to effectively treat arteriovenous malformations in the oral region without any significant morbidity.

## 1. Introduction

“Vascular malformation” is a generalized term used for the description of a set of congenital errors during vascular development, due to anomalous proliferation of vascular (angio/lympho) structures. This condition occurs in about one percent of births and is often very mild and does not require therapy [1]. Currently, Mulliken and Glowacki's 1982 classification system is still held as a standard for vascular anomalies. The classification system is based on the parameters that include the clinical, histopathological presentation, and biological behavior [2, 3]. Arteriovenous malformations (AVMs) are categorized as malformations with a high-flow capability as they involve arterovenous communications without the intermediate capillary network [4]. Also, the proliferation of AVM is induced by several other factors such as ischemia secondary to thrombosis, puberty, changes in hormonal levels, trauma, and ectasia. In the case of trauma, only a single vessel is involved in AVM; however, when the condition is familial, multiple vessels are involved [5, 6].

The head and neck are one of the most common sites (nearly 60% cases) for AVM [7]. 4 to 5% of all craniofacial AVMs occur in the gnathic bones. AVMs of the gnathic bones are relatively harder to treat [4]. Familial AVMs are exceedingly rare. RASA1 gene mutation expressing p120-Ras GAP, on chromosome 5q, has been reported to be associated with familial congenital AVM [8]. Most cases of familial AVMs are noted at birth although childhood or adolescence manifestations are not uncommon. Rarely, AVMs can associate with high output cardiac failure and cardiomegaly. The lesions progress gradually and have can potentially manifest in any body part including the oral cavity. Within the oral cavity, the tongue (anterior two-thirds), gingival, palate, and buccal mucosa are the most frequently reported sites of occurrence [9]. Kohout et al. has reported the largest case series of 81 cases of AVM that manifested in the oral cavity. In their case series, 31% occurred in the cheek (31%), 16% in the ears, 10% in the nose and forehead each, 7% in the upper labial mucosa, 5% each in the mandible and neck, and 4% each in the scalp and maxilla [5].

Clinically, these lesions occur as a pulsatile mass with a thrill, bruit, and rarely local hyperthermia with bleeding. There is a decreased vascularity leading to decreased nutrition supply resulting in necrosis, ulceration, bleeding, and impairment of function. The skin over the lesions is seen to have a reddish or true port-wine hue [10, 11].

Considering the diagnosis of the condition, the most common diagnostic modalities include radiography, CT (computed tomography scans), MRI (magnetic resonance imaging), and angiography [12]. Given the complex anatomical pathology, the treatment strategy for an AVM often involves multispecialties. The most common conventional therapeutic modalities suggested for AVM are a combination of surgical resection, sclerotherapy, and endovascular embolization techniques [12]. Ramakrishnan et al. in their systematic review have reported that the management of various vascular malformations included AVM. In their review, 6 articles reported various management techniques of AVM [13]. Deng et al. had reported Superselective Intra-Arterial Embolization (SIAE) followed by curettage (CT) or surgical resection (SR) for the management of AVM with complete involution and favorable prognosis [14].

SIAE and CT followed by sclerotherapy with complete involution and absence of recurrence was reported by Chen et al. [15]. Chen et al. reported 60.7% involution with recurrence and complications associated with sclerotherapy and fractionated radiotherapy [16]. Fan et al. reported 80% involution associated with complications in direct puncture embolization [17], and Zhao et al. reported 100% involution and nil recurrence with sclerotherapy alone [18]. Behnia et al. reported that curettage via proximal osteotomy without complete resection is an effective and less invasive method with more esthetics and better function in comparison with surgical resection [19].

Recently, LASERs, especially CO_2_ and Er, Cr:YSGG, have been reported to be a successful tool for the treatment of AVM cases [20]. In the present paper, we present the minimally invasive surgical management of familial soft tissue AVM of the oral cavity reported in siblings.

## 2. Case Reports

### 2.1. 1^st^ Case

**Figure 1:**
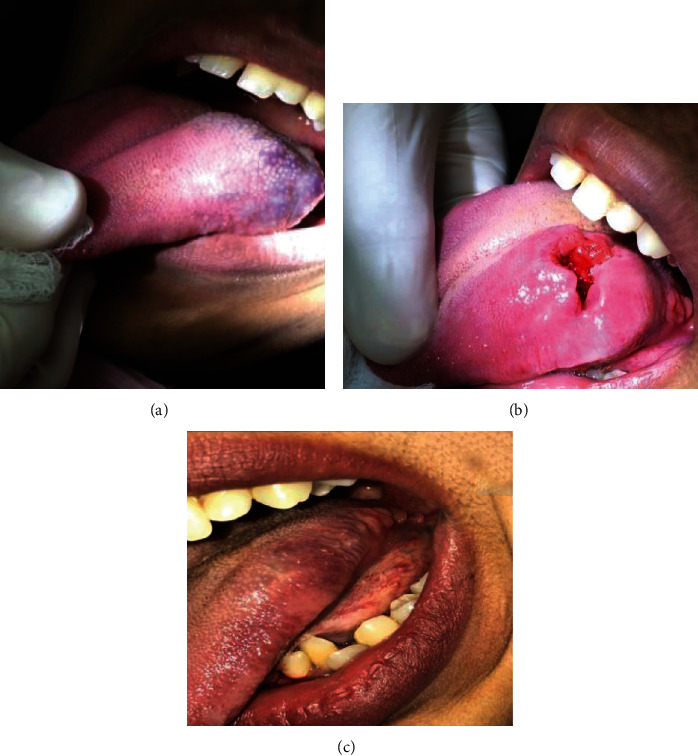
(a) Preoperative AVM primary lesion of the left posterior region of the tongue; (b) AVM primary lesion opened after being injected 2 times; (c) follow-up after complete resolution of the primary AVM lesion by 3 years.

A female patient 16 years of age reported to the Department of Oral and Maxillofacial Surgery of the College of Dentistry, Jazan University, Jazan, Saudi Arabia. The patient's medical history did not reveal any significant findings. The primary complaint of the patient was a persistent swelling of 10-year duration in the tongue. The swelling was not associated with pain and had been increasing in size gradually. There were no other symptoms associated with the swelling. On examination, the lesion was multilobulated and bluish with intact overlying mucosa and was present along the left lateral border and dorsum of the tongue opposite the mandibular left second molar. The lesion measured 4 cm by 6 cm by 0.7 cm (Figure 1(a)).

The consistency of the swelling was soft. Palpation did not reveal any tenderness. On compression, the swelling blanched. The swelling did not have any significant pulsation. All hematological parameters were within the physiological limit. Potential bone involvement was ruled out with the aid of plain radiographs. The AVM diagnosis was inferred using the findings from the MRI.

**Figure 2:**
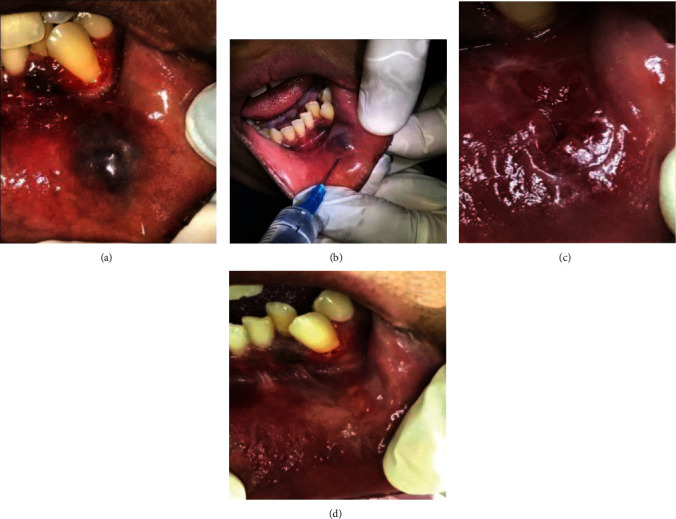
(a) Second lesion which appeared after the tongue lesion had resolved by 2 years; (b) boiling saline injection of the second AVM lesion; (c) second AVM lesion on the way to healing after 3 boiling saline injections; (d) second AVM lesion after 1 year of complete healing.

The patient was advised conservative surgical management using boiling saline injection. The procedure and possible complications were explained to the patient, and written informed consent was obtained. The patient was injected with 5 ccs of boiling saline after she was given a lingual nerve block, then the pressure was applied to the lesion to control bleeding. The patient was given postoperative instructions and recalled after two weeks. The same procedure was repeated at the recall appointment. One week later, the lesion was examined, and fibrosis was observed along with an open wound on the surface at the center of the lesion (Figure 1(b)). The patient was prescribed Levofloxacin Tab 500 mg (Sanofi Winthrop industries, 56, route de Choisy au Bac, 60205 Compiegne, France), one tablet a day for 5 days, and Ibuprofen Tab 200 mg (Wyeth Pharmaceuticals Company, Guayama, PR, USA), 2 tablets twice a day for 5 days. The lesion had healed uneventfully three months after the first injection (Figure 1(c)). The patient was followed up for three years, and the tongue lesion was free from any symptoms or recurrence. However, two years into the treatment of the tongue lesion, another arteriovenous malformation at the labial mucosa on the left side opposite the left lower canine tooth was noticed. The lesion was about 4 × 3 cm in size and about 0.5 cm in height (Figure 2(a)). The patient was administered the first boiling saline injection after giving her a mental nerve block (Figure 2(b)).

The saline (4 ccs) was injected around the lesion from different aspects while pointing the needle towards the depth of the lesion. For this lesion, the patient was injected with boiling saline on two other occasions that are two and three months after the first injection. The lip lesion started healing uneventfully four months after the first appointment (Figure 2(c)). There were no signs of any complications or recurrence at the six-month and one-year follow-up (Figure 2(d)).

### 2.2. 2^nd^ Case

**Figure 3:**
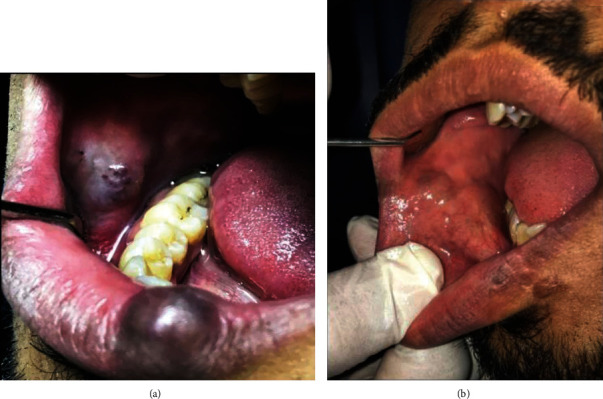
(a) Preoperative photograph of both lip and cheek AVM lesions; (b) postoperative photograph of both lip and cheek AVM lesions.

The brother of the patient in case 1 was a 23-year-old who used to visit the department with his sister for her appointments. The patient had two AVMs. The first lesion was on the lower vermillion border on the left side and measured 2.5 cm by 1.5 cm. The lesion led to the visible asymmetry of the lower lip. The second lesion was present on the right buccal mucosa opposite to the first lower right molar and extending to the canine tooth, measuring about 4 × 3 cm (Figure 3(a)). The lesions were not associated with pain and had been increasing in size gradually. On examination, the lesion was multilobulated and bluish. The examination did not reveal any discontinuity in the surface mucosa. As reported for the 1^st^ case, palpation revealed that the lesion was soft and nontender and on compression exhibited blanching. No significant pulsation was noted. Hematological parameters were all within the physiological limit. Bone involvement was ruled out with plain radiographs. AVM diagnosis was inferred with the findings from the MRI. Citing cosmetic concerns, treatment was sought for the lesion. After obtaining informed consent, the same treatment plan as used for the sister was advised to the patient. He was injected with 4 ccs of boiling saline around the lip lesion and at different depths under the mental nerve block. The pressure was applied to the lesion to control bleeding. The patient was given postoperative instructions and recalled after two weeks.

The patient was given three additional injections at one week, four weeks, and six weeks after the first injection. Additionally, the patient was prescribed Panthenol 10% gel (Pamex Pharmaceuticals, Germany) for application on the skin of the lip. The healing was uneventful with no complication or recurrence at the three-month and six-month appointments (Figure 3(b)).

## 3. Discussion

AVMs frequently noted at birth can manifest in childhood/adolescence [1, 9]. In the present cases, the patients had a history of the AVMs for about ten years and have been constantly growing in size. Kohout et al. reported the location of 81 AVMs in the head and neck. The cheek (31%) was the most common site followed by the ear (16%), nose (10%), upper lip (7%), mandible (5%), neck (5%), scalp (5%), and maxilla (4%) [8]. Within the oral cavity, the AVM's most frequent site is the tongue as in the present case. The lesser-known sites for intraoral AVM include the gingiva, palate, and buccal mucosa. AVM can be a major hurdle during speech, mastication, and deglutition and often get traumatized resulting in ulceration and secondary infection [21, 22]. In our cases, one lesion was on the tongue and two were on the lip (one on the labial mucosa and one on the vermillion border). The patients did not report any symptoms associated with the lesions and decided to undergo treatment for aesthetic reasons only.

Management of AVMs has always posed a challenge among clinicians because of their unpredictable behavior and high recurrence rate [23, 24]. Over the years, a plethora of approaches, including surgical excision, irradiation, electrocoagulation, cryotherapy, intravascular magnesium or copper needles, systemic corticosteroids, interferon-*α*, embolization, lasers, and sclerotherapy, has been tried for the management of these lesions. However, there is no universally accepted model of treatment for AVMs [25–27]. Moreover; each method has its limitations and disadvantages. Surgical excision, owing to the complicated anatomy of the face and neck, may result in significant loss of function, aesthetic issues, hemorrhage, or nerve damage [28]. Laser therapy is expensive. Additionally, it may lead to scarring and hyperpigmentation. Similar issues have also been noted with cryotherapy [29, 30]. Corticosteroids have systemic effects like adrenal suppression and weight gain [31].

Sclerotherapy is a conservative and low-cost technique for the effective treatment of vascular lesions of benign nature, including AVMs. It is particularly advantageous in surgically inaccessible areas. Sclerotherapy causes minimal scarring and has relatively fewer complications as compared to surgical excision [32]. Many sclerosing agents, for example, 5% sodium morrhuate, 5% ethanolamine oleate, sodium phyllite, 1% polidocanol, sodium tetradecyl sulfate, quinine urethane, hypertonic saline, and absolute ethanol, alone or in combination, have been used with varying results [24, 27].

In the present cases, boiling saline was used as the sclerosing agent for the treatment. The heat released from the boiling saline dehydrates the endothelial and red blood corpuscles, causing necrosis-induced vessel lumen obliteration, which in turn augments blood coagulation and vessel death. Additionally, it is readily available, acceptable to the patients as it is not a chemical, hypoallergenic, and inexpensive [33–35].

Sclerotherapy has certain potential complications like ulcerations, swelling, infection, transient nerve damage, hemorrhage, and anaphylaxis [35]. However, none of the aforementioned complications were observed in our patients. Eltohami and Abuaffan used hot saline for the treatment of a venous malformation in the tongue with success [33]. Water and hypertonic saline have also been used as means for sclerotherapy in venous malformations by Obuekwe et al. [36] and Oji et al. [37].

## 4. Conclusion

Arteriovenous malformations of the oral cavity might be associated with several complications apart from being an aesthetic concern. Therefore, prompt diagnosis and treatment are warranted for such lesions. Sclerotherapy with boiling saline provides an effectively safe therapeutic modality for intraoral AVMs. This method resulted in complete remission and alleviation of their symptoms, with no complications. No recurrence of the lesions was observed during the follow-up period.
